# Prognostic Value of Tumor Regression Grade in Geriatric Patients with Locally Advanced Gastric Adenocarcinoma

**DOI:** 10.3390/medicina62091668

**Published:** 2026-08-31

**Authors:** Didar Senocak, Bunyamin Guney, Mina Karakullukcu, Oguz Bilgi, M. Alpaslan Ozgun

**Affiliations:** 1 Department of Medical Oncology, Sultan II. Abdulhamid Han Training and Research Hospital, University of Health Sciences, Istanbul 34668, Türkiye; mina.zoralioglu@gmail.com (M.K.); ogzblg@yahoo.com (O.B.); alpozgun@yahoo.com (M.A.O.); 2 Department of Medical Oncology, Istanbul Training and Research Hospital, University of Health Sciences, Istanbul 34098, Türkiye; drbunyaminguney01@gmail.com

**Keywords:** geriatric patients, gastric adenocarcinoma, Becker tumor regression grading, pathological response, disease-free survival, overall survival

## Abstract

*Background and Objectives*: Tumor regression grade (TRG) in gastric cancer patients treated with neoadjuvant/perioperative chemotherapy is increasingly recognized as an important indicator of treatment efficacy and long-term prognosis. However, in geriatric patients, data on neoadjuvant therapy and on the relationship between pathological response and survival remain limited. In this study, we investigated the prognostic significance of pathological response, assessed according to Becker’s TRG in geriatric patients with locally advanced gastric adenocarcinoma who received neoadjuvant/perioperative chemotherapy and underwent gastrectomy. *Materials and Methods*: We retrospectively analyzed 82 patients aged ≥65 years with histologically confirmed gastric adenocarcinoma who received neoadjuvant/perioperative chemotherapy followed by curative gastrectomy at a single center between 2017 and 2024. Pathological response was evaluated using the Becker tumor regression grading (TRG) system and classified as good (TRG 1–2) or poor (TRG 3). Clinicopathological features, treatment characteristics, disease-free survival (DFS), and overall survival (OS) were compared between groups. Survival was estimated using the Kaplan–Meier method and compared with the log-rank test. Univariable and multivariable Cox proportional hazards models were used to assess the prognostic impact of TRG and other clinicopathological factors on DFS and OS. *Results*: The study included 82 geriatric patients, of whom 50 (61.0%) showed a good pathological response (TRG 1–2), while 32 (39.0%) showed a poor pathological response (TRG 3). Poor response was more frequently seen in clinical stage III disease and was accompanied by a higher rate of lymphovascular invasion. Median DFS was 42.4 months in the good response group versus 13.0 months in the poor response group (*p* < 0.001), and median OS was 47.7 and 15.5 months, respectively (*p* < 0.001). In multivariable analysis, ECOG performance status (HR 2.697; 95% CI 1.381–5.269; *p* = 0.004), lymphovascular invasion (HR 4.960; 95% CI 1.545–15.919; *p* = 0.007), and poor pathological response (HR 2.066; 95% CI 1.055–4.045; *p* = 0.034) remained independent predictors of DFS. For OS, ECOG performance status (HR 2.719; 95% CI 1.398–5.290; *p* = 0.003), lymphovascular invasion (HR 4.448; 95% CI 1.400–14.132; *p* = 0.011), and poor pathological response (HR 2.311; 95% CI 1.161–4.601; *p* = 0.017) remained independent predictors of OS. *Conclusions*: Our study suggests that pathological response assessed by the TRG system is a valuable prognostic marker in geriatric gastric adenocarcinoma. Good pathological response was associated with longer DFS and OS, while poor pathological response remained an independent predictor of both shorter DFS and OS.

## 1. Introduction

Gastric cancer is the fifth most commonly diagnosed malignancy worldwide and remains a major global health problem. In 2022, nearly one million new cases were reported, accounting for approximately 6.8% of all cancer-related deaths [1]. Although its incidence has been declining, particularly in developed countries, the absolute number of gastric cancer cases is expected to increase due to demographic changes, population growth, and longer life expectancy. With the aging of the global population, a substantial proportion of patients diagnosed with gastric cancer in the coming years will be geriatric, making the definition of optimal treatment strategies in this group increasingly important [2]. Gastric cancer is especially prevalent in East Asia and is more common in men than in women. The highest incidence is observed in the seventh decade of life [3]. Geriatric patients, however, represent a heterogeneous population, not only in terms of chronological age but also with respect to functional capacity and comorbidity burden [4]. There are no surveillance or treatment guidelines specifically tailored to geriatric age groups, which complicates decisions regarding the upper age limit for screening, diagnostic procedures, and invasive treatments. Approximately two-thirds (60–70%) of patients are diagnosed at a locally advanced stage, for which chemotherapy, immunotherapy, and surgery constitute the main treatment modalities. In geriatric gastric cancer patients, an additional factor that may increase disease-related mortality is the reluctance of both older adults and clinicians to perform or recommend standard diagnostic and therapeutic interventions because of concerns about complications [5].

The major limiting factors in geriatric gastric cancer include increased disease burden in the presence of multiple comorbidities, malnutrition, frailty, and reduced performance status. Comorbid conditions such as cardiovascular disease, diabetes, and chronic kidney disease can increase treatment-related toxicity, decrease treatment completion rates, and heighten the risk of adverse effects. Geriatric patients are also under-represented in clinical trials, particularly in perioperative treatment studies; consequently, much of the available evidence is derived from younger patients with good performance status. These issues create uncertainty in treatment selection for geriatric patients and may result in some individuals not receiving standard therapies or being treated with insufficient dose intensity [6]. Perioperative treatment is now a key component of the standard approach for locally advanced gastric cancer, aiming to reduce tumor burden and increase the chance of curative resection. Although pathological response after treatment has been linked to long-term survival, data on factors predicting pathological response specifically in the geriatric population are limited [7]. Identifying factors that influence treatment response in geriatric gastric cancer patients, selecting appropriate candidates, and individualizing therapy are therefore crucial. This study aimed to evaluate the outcomes of perioperative treatment in geriatric patients (≥65 years) with locally advanced gastric cancer, to determine pathological treatment response, to identify clinical, laboratory, and pathological factors associated with response, and to investigate the impact of these factors on survival.

## 2. Materials and Methods

### 2.1. Study Design

This single-center, retrospective study included geriatric patients with locally advanced gastric cancer who were followed in the Department of Medical Oncology at Sultan II. Abdulhamid Han Training and Research Hospital, University of Health Sciences. The study was approved by the Non-Interventional Clinical Research Ethics Committee of Sultan II. Abdulhamid Han Training and Research Hospital (Decision No: 2026-122). Patients aged 65 years and older who were diagnosed histopathologically with locally advanced gastric adenocarcinoma and underwent curative gastrectomy after perioperative treatment between 1 January 2017 and 31 December 2025 were included. As perioperative chemotherapy, patients received either fluorouracil, leucovorin, oxaliplatin, and docetaxel (FLOT) or fluorouracil, leucovorin, and oxaliplatin (FOLFOX). The FLOT regimen consisted of docetaxel (50 mg/m^2^), oxaliplatin (85 mg/m^2^), leucovorin (200 mg/m^2^), and 5-fluorouracil (2600 mg/m^2^) administered as a 24 h continuous intravenous infusion every 2 weeks, with four preoperative cycles planned. The FOLFOX regimen consisted of oxaliplatin (85 mg/m^2^), leucovorin (200 mg/m^2^), 5-fluorouracil (400 mg/m^2^ as an intravenous bolus), followed by 5-fluorouracil (2400 mg/m^2^) administered as a 46 h continuous intravenous infusion every 2 weeks, with six preoperative cycles planned. The treatment regimen was selected according to the patient’s age, comorbidities, performance status, treatment tolerance, and prevailing clinical practice at the time of treatment. Following neoadjuvant treatment, patients considered suitable for curative surgery underwent gastrectomy with D2 lymph node dissection. In the postoperative period, completion of perioperative chemotherapy was planned for clinically eligible patients who were able to tolerate further treatment, with four postoperative cycles of FLOT or six postoperative cycles of FOLFOX, according to the regimen initially administered.

**Figure 1:**
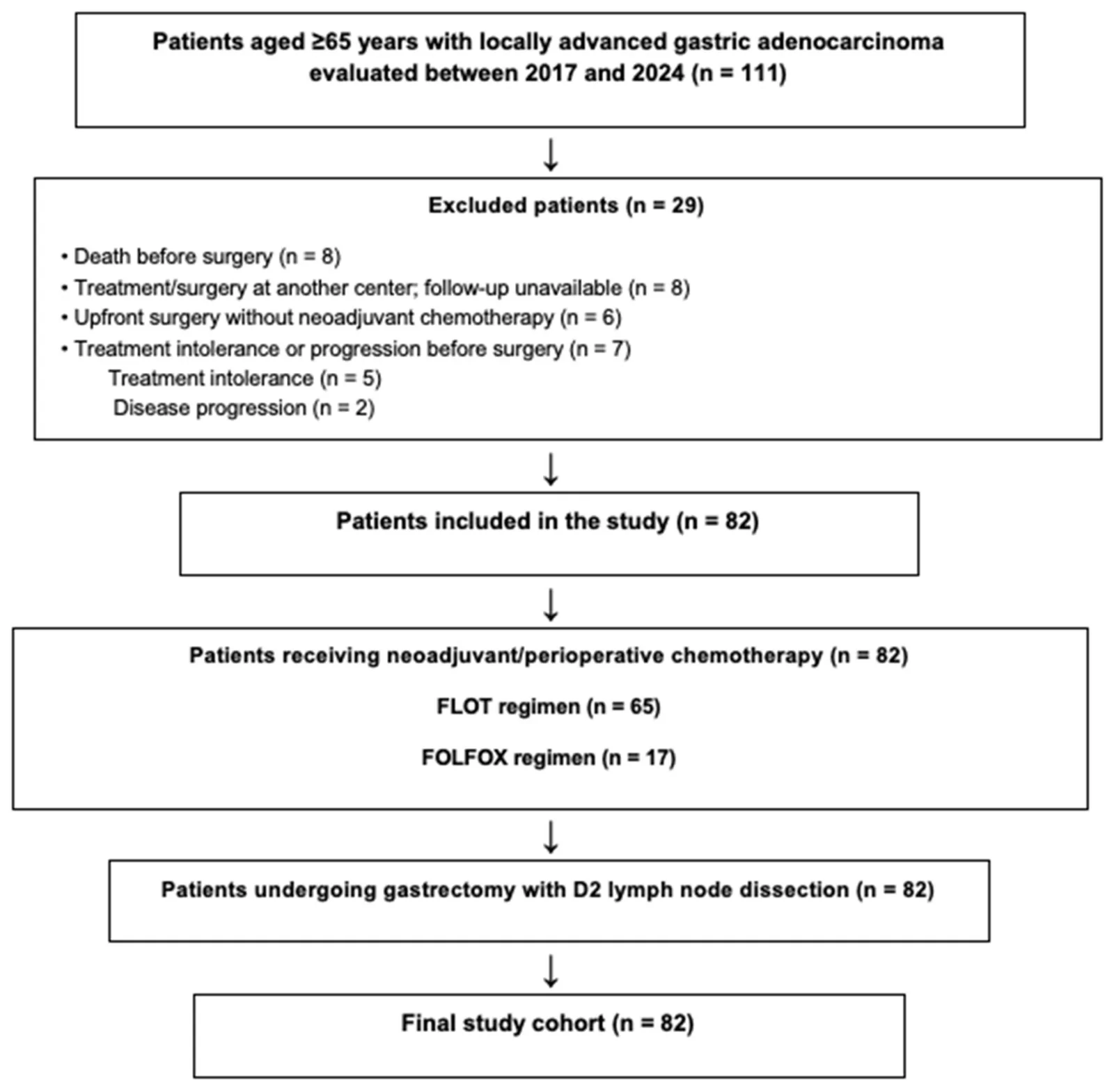
Flowchart of patient selection and study cohort.

During the study period, 111 patients aged ≥65 years with locally advanced gastric adenocarcinoma were assessed for eligibility. After exclusion of patients who did not meet the study criteria or did not proceed to curative surgery, 82 patients constituted the final study cohort. Among the 89 patients who initiated neoadjuvant chemotherapy, 5 (5.6%) discontinued treatment because of treatment intolerance and did not proceed to surgery. The detailed patient selection process is presented in Figure 1. Inclusion criteria were age ≥65 years at diagnosis, histologically confirmed locally advanced gastric adenocarcinoma, and having undergone curative-intent surgery. Clinical stage was determined before the initiation of neoadjuvant chemotherapy based on pretreatment clinical and radiological assessments and was considered a baseline clinical characteristic in the analyses. Pathological treatment response was evaluated on the surgical specimen according to the Becker tumor regression grading (TRG) system. In this classification, TRG 1 indicates complete or subtotal regression (<10% residual tumor), TRG 2 partial regression (10–50% residual tumor), and TRG 3 minimal or no regression (>50% residual tumor). Patients with TRG 1 or 2 were grouped as having a good pathological response, whereas those with TRG 3 were classified as having a poor pathological response. Pathological complete response (pCR) was defined as the absence of residual invasive tumor in the surgical specimen (ypT0N0). After surgery, patients were regularly followed with clinical assessments, laboratory tests, and imaging studies, primarily computed tomography (CT). Follow-up evaluations were performed every 3 months during the first 2 years and every 6 months thereafter. Disease recurrence or progression was determined based on clinical and radiological assessments. DFS was defined as the time from the date of surgery to the date of disease recurrence, progression, or death from any cause, whichever occurred first. OS was defined as the time from the date of diagnosis to death from any cause.

### 2.2. Statistical Analysis

Descriptive statistics for the data included mean, standard deviation, median, frequency, and percentage values. The distribution of the variables was assessed using the Kolmogorov–Smirnov and Shapiro–Wilk tests. Quantitative independent variables that did not show a normal distribution were analyzed with the Mann–Whitney U test. Categorical independent variables were analyzed with the chi-square test, and when the assumptions of the chi-square test were not met, Fisher’s exact test was used. Survival analysis was performed using the Kaplan–Meier method. Comparisons of survival curves were made with the log-rank test. Univariable Cox regression analysis was performed for variables showing clinically or statistically significant differences between the groups. Multivariable Cox regression analysis was subsequently performed to account for potential confounding factors, and the results were reported as hazard ratios (HRs) with 95% confidence intervals (CIs). All statistical analyses were conducted with IBM SPSS Statistics, version 28.0 (IBM Corp., Armonk, NY, USA). A *p* value of <0.05 was considered statistically significant.

## 3. Results

A total of 82 patients were included in the study. The mean age of the patients was 69.3 ± 4.8 years, and male patients accounted for 75.6% of the cohort. The ECOG performance status was 0–1 in 97.6% of the patients. The most frequent comorbidities were hypertension (41.5%) and diabetes mellitus (26.8%). Histopathological evaluation revealed poorly differentiated adenocarcinoma in 54.9% of cases, and the most common primary tumor locations were the cardia (39.0%) and the antrum (30.5%) (Table 1).

Fifty-four patients (65.9%) had clinical stage III disease. With respect to perioperative treatment, 65 patients (79.3%) received the FLOT regimen, and 17 (20.7%) received the FOLFOX regimen. The median number of neoadjuvant chemotherapy cycles was 4 (range, 3–6), and the median number of adjuvant chemotherapy cycles was 4 (range, 0–6). Total gastrectomy was performed in 49 patients (59.8%) and subtotal gastrectomy in 33 patients (40.2%). Lymphovascular invasion was detected in 57 patients (69.5%), and perineural invasion in 53 patients (64.6%). Pathological complete response (pCR) was achieved in three patients (3.7%). According to TRG, 50 patients (61.0%) had a good pathological response (TRG 1–2), whereas 32 patients (39.0%) had a poor pathological response (TRG 3). During follow-up, disease progression was observed in 30 patients (36.6%). Of the 30 patients who developed progression, 17 (56.7%) received second-line systemic therapy. During the follow-up period, 44 patients (53.7%) died (Table 2). When the Becker tumor regression grades were presented separately, TRG 1 was observed in 5 (6.1%) patients, TRG 2 in 45 (54.9%) patients, and TRG 3 in 32 (39.0%) patients.

When the good and poor pathological response groups were compared, no statistically significant differences were observed in age at diagnosis, sex, primary tumor location, or type of surgery (*p* > 0.05 for all variables). Lymphovascular invasion was significantly more frequent in the poor pathological response group (90.6%) than in the good pathological response group (56.0%) (*p* = 0.001), whereas the frequency of perineural invasion was similar between the groups (75.0% versus 58.0%; *p* = 0.116). Clinical stage III disease was significantly more common in the poor pathological response group than in the good pathological response group (93.8% versus 48.0%; *p* < 0.001). Regarding perioperative chemotherapy regimen, there was no significant difference between the good and poor pathological response groups (FLOT: 76.0% versus 84.4%; FOLFOX: 24.0% versus 15.6%; *p* = 0.361). In contrast, disease progression was significantly more frequent in the poor pathological response group than in the good pathological response group (50.0% versus 28.0%; *p* = 0.044). Similarly, the mortality rate was significantly higher in the poor pathological response group (75.0% versus 40.0%; *p* = 0.002) (Table 3).

**Figure 2:**
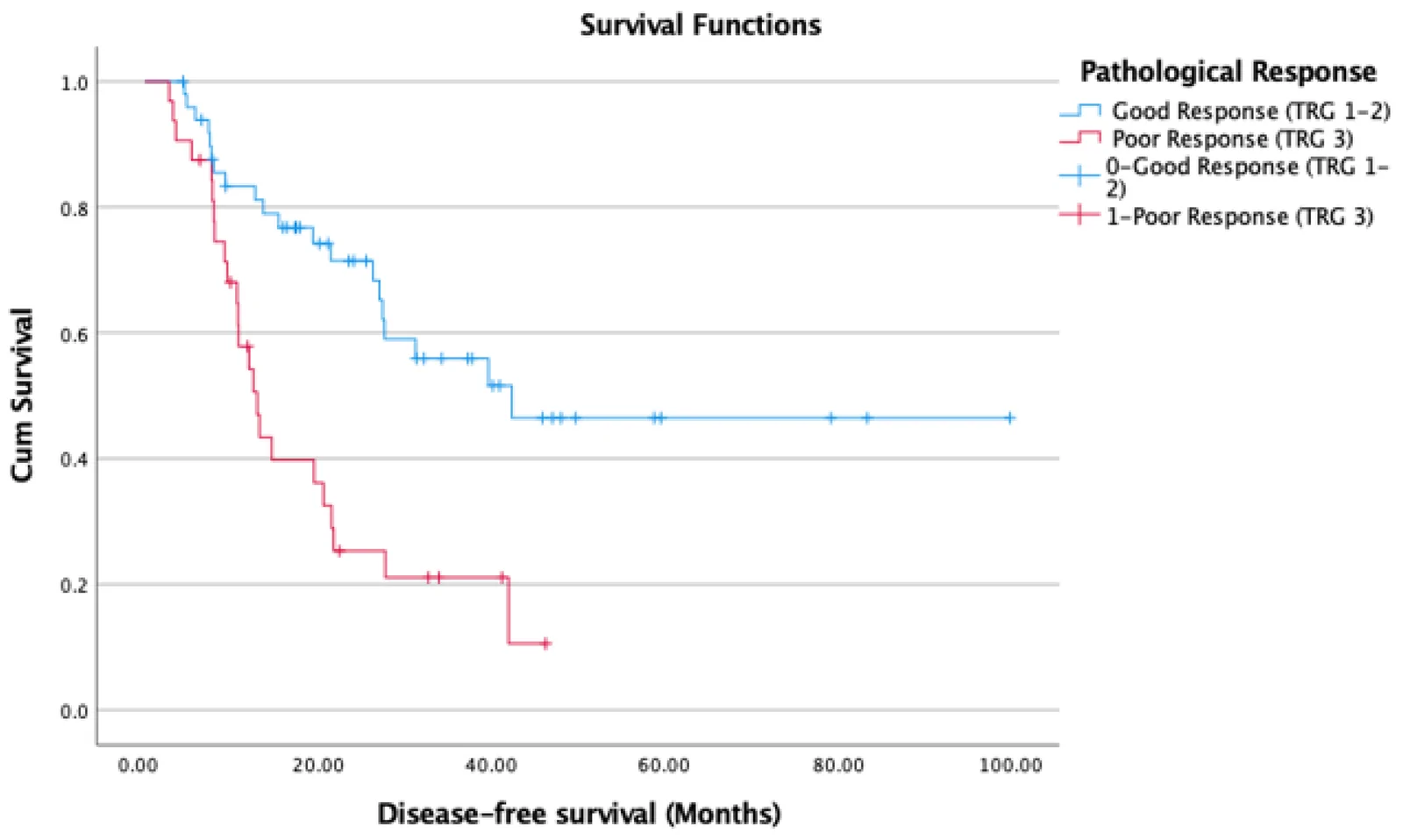
Kaplan–Meier curves of DFS according to pathological response.

Kaplan–Meier analysis demonstrated significantly longer disease-free survival (DFS) in patients with a good pathological response (TRG 1–2) compared with those with a poor pathological response (TRG 3) (Figure 2). The median DFS was 42.4 months (95% CI, 29.3–56.1) in the good-response group and 13.0 months (95% CI, 9.9–16.0) in the poor-response group (*p* < 0.001) (Table 4).

**Figure 3:**
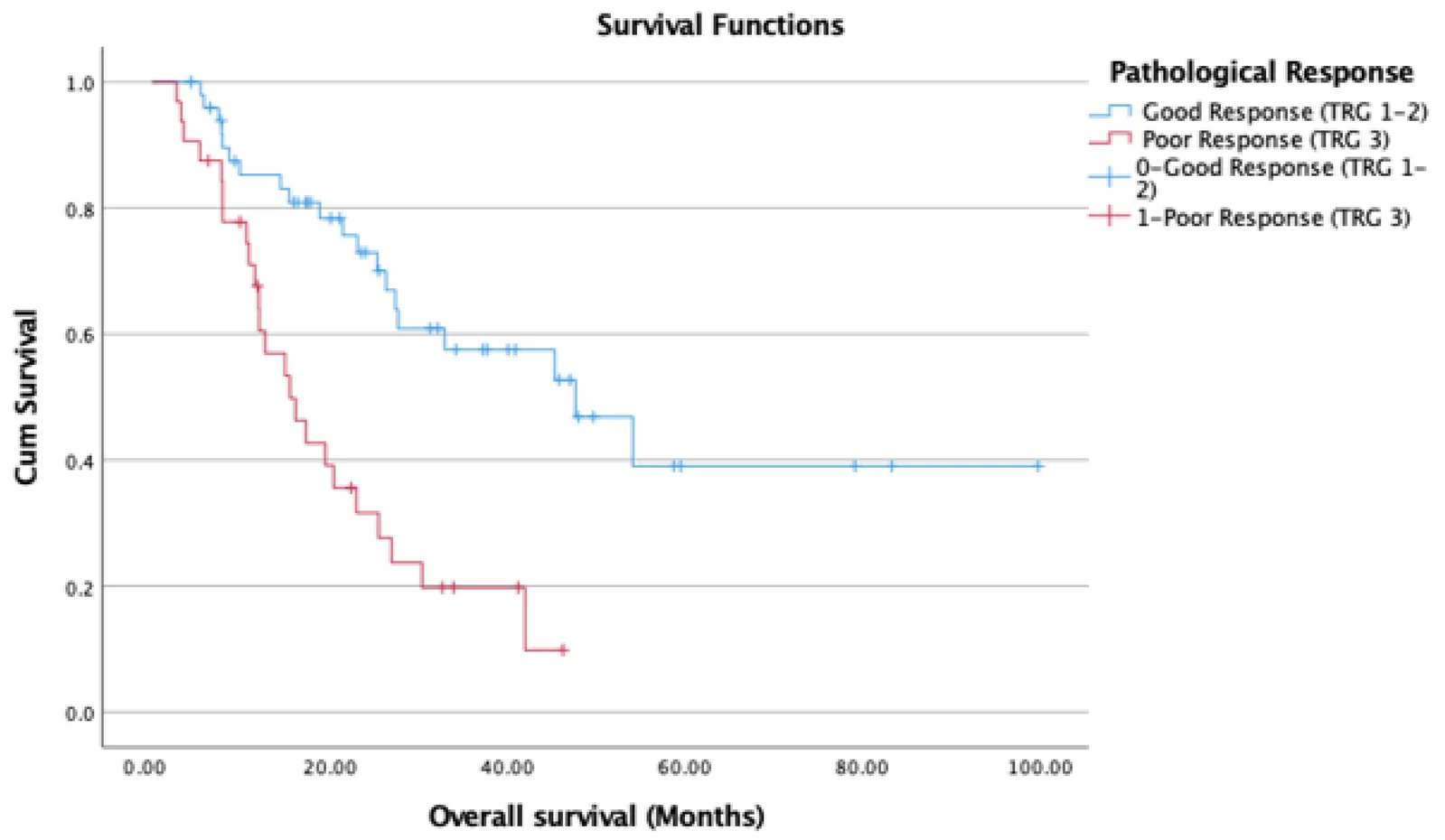
Kaplan–Meier curves of OS according to pathological response.

Kaplan–Meier analysis demonstrated significantly longer overall survival (OS) in patients with a good pathological response (TRG 1–2) compared with those with a poor pathological response (TRG 3). Median OS was 47.7 months (95% CI, 26.2–69.2) in the good-response group and 15.5 months (95% CI, 9.6–21.4) in the poor-response group (log-rank *p* < 0.001) (Figure 3).

For DFS, univariable Cox regression analysis showed statistically significant associations between DFS and ECOG performance status, clinical stage, lymphovascular invasion, and poor pathological response (TRG 3). ECOG performance status was associated with a 3.1-fold increased risk of recurrence or death (HR: 3.102; 95% CI: 1.601–6.012; *p* < 0.001), while clinical stage III was associated with a 2.2-fold increased risk compared with stage II (HR: 2.188; 95% CI: 1.078–4.440; *p* = 0.030). The presence of lymphovascular invasion was associated with a 6.4-fold increased risk of recurrence or death (HR: 6.433; 95% CI: 2.271–18.220; *p* < 0.001). Similarly, in patients with poor pathological response (TRG 3), the risk of recurrence or death was 2.9 times higher than in those with good pathological response (TRG 1–2) (HR: 2.893; 95% CI: 1.581–5.296; *p* < 0.001). In multivariable Cox regression analysis, ECOG performance status, lymphovascular invasion, and TRG 3 remained significant independent prognostic factors for DFS. ECOG performance status was associated with an approximately 2.7-fold higher risk of recurrence or death (HR: 2.697; 95% CI: 1.381–5.269; *p* = 0.004), lymphovascular invasion with an approximately 5.0-fold higher risk (HR: 4.960; 95% CI: 1.545–15.919; *p* = 0.007), and TRG 3 with an approximately 2.1-fold higher risk compared with TRG 1–2 (HR: 2.066; 95% CI: 1.055–4.045; *p* = 0.034) (Table 5).

For OS, univariable Cox regression analysis showed statistically significant associations between OS and ECOG performance status, clinical stage, lymphovascular invasion, and poor pathological response. ECOG performance status was associated with an approximately 3.1-fold higher risk of death (HR: 3.106; 95% CI: 1.613–5.980; *p* < 0.001), clinical stage III disease with an approximately 2.3-fold higher risk (HR: 2.346; 95% CI: 1.144–4.812; *p* = 0.020), lymphovascular invasion with an approximately 6.3-fold higher risk (HR: 6.341; 95% CI: 2.248–17.885; *p* < 0.001), and poor pathological response with an approximately 3.2-fold higher risk (HR: 3.239; 95% CI: 1.737–6.040; *p* < 0.001). In multivariable Cox regression analysis, ECOG performance status (HR: 2.719; 95% CI: 1.398–5.290; *p* = 0.003), lymphovascular invasion (HR: 4.448; 95% CI: 1.400–14.132; *p* = 0.011), and poor pathological response (HR: 2.311; 95% CI: 1.161–4.601; *p* = 0.017) remained significant independent prognostic factors for OS (Table 6).

## 4. Discussion

Gastric cancer is one of the malignancies whose incidence increases markedly in older adults in parallel with the expansion of the geriatric population, and this makes the management of older patients increasingly important. In elderly patients, the frequent presence of comorbidities, reduced physiological reserves, and concerns regarding treatment-related toxicities often render the clinical management of geriatric cancer patients challenging. As a consequence of these factors, this patient group is under-represented in prospective clinical trials, and the generalizability of the available data to the real-world geriatric population remains limited [8]. In patients with locally advanced gastric cancer, most of the current evidence on the prognostic significance of pathological response after neoadjuvant/perioperative treatment, which has become the standard of care, has been derived from younger or age-unselected populations, and data specifically focusing on geriatric patients are still limited [9]. Perioperative treatment with the FLOT regimen has been associated with significant improvements in survival outcomes. However, in real-world practice, intensive FLOT therapy may not be suitable for all patients due to factors such as age, comorbidity burden, and performance status; in such cases, alternative regimens with better tolerability (for example, FOLFOX) are preferred, and geriatric cohorts treated with these regimens have also been reported [10]. However, it is known that treatment success depends not only on the chemotherapy regimen administered but also on the degree of the tumor’s biological response to systemic therapy. In this context, the TRG assessed after neoadjuvant treatment is considered a strong pathological marker for predicting long-term oncologic outcomes; in particular, the Becker-based TRG classification is widely used in both clinical studies and routine practice because it reflects the amount of residual tumor in a relatively objective manner [11].

In our study, we evaluated the prognostic significance of pathological response in geriatric patients with gastric adenocarcinoma who received perioperative chemotherapy followed by curative surgery. Good pathological response (TRG 1–2) was associated with significantly longer DFS and OS than poor pathological response (TRG 3), supporting the prognostic value of the TRG system in this patient population. The rate of pathological complete response (pCR) was 3.7%. Many studies have shown that pCR rates vary according to the chemotherapy regimen used, tumor stage, and patient population. In the seminal study by Becker and colleagues, in which they first described the tumor regression grading system, patients received neoadjuvant therapy with a combination of etoposide, doxorubicin, and cisplatin (EDP). Among the 36 patients evaluated, complete regression was not observed in any case, and the proportion of patients with <50% residual tumor—corresponding to the good response category in our study—was 36%, which is lower than the 61% observed in our cohort. This difference may be related to the use of more effective perioperative regimens in current practice, as well as differences in patient selection and stage distribution [12]. In the phase II analysis of the FLOT4-AIO trial, the rate of complete histopathological regression with the 5-fluorouracil, leucovorin, oxaliplatin, and docetaxel (FLOT) regimen was 16% in the FLOT arm, whereas the corresponding rate with epirubicin, cisplatin, and 5-fluorouracil or capecitabine (ECF/ECX) was 6%; moreover, the rate of major pathological regression was shown to be approximately twofold higher in favor of the FLOT regimen [13]. In our study, the pCR rate was lower than that reported in the FLOT4-AIO trial, which can primarily be explained by differences in patient populations between the two studies. While FLOT4-AIO was conducted under clinical trial conditions and included younger, highly selected patients with better performance status, our study reflects real-world data from patients aged 65 years and older with additional comorbidities. Furthermore, in our cohort, 20% of patients did not receive FLOT but were treated with FOLFOX instead. The high rate of good pathological response (61%) observed in our study indicates that, even in geriatric patients, meaningful tumor regression can be achieved in a substantial proportion of cases, despite the low pCR rate, and underscores that the effectiveness of neoadjuvant therapy in older adults should not be assessed solely on the basis of pCR.

In our analysis, the proportion of stage III disease in the poor pathological response (TRG 3) group was 93.8%, and the frequency of lymphovascular invasion (LVI) was 90.6%, whereas in the good pathological response (TRG 1–2) group these values were 48.0% and 56.0%, respectively. These findings suggest that insufficient tumor regression may be associated with a higher tumor burden and more aggressive tumor biology. Consistent with our results, Xie et al. reported that poor tumor regression grade after neoadjuvant chemotherapy in gastric cancer was associated with advanced pathological stage and worse long-term oncologic outcomes [14]. In another study of gastric cancer patients, LVI was shown to be associated with increased lymph node metastasis, more advanced disease stage, and to be an independent adverse prognostic factor for both DFS and OS [15]. Similarly, in the study by Zhang et al., which evaluated 1488 patients with stage I–III gastric cancer, LVI positivity was identified as a poor prognostic factor for OS [16]. Taken together, these data indicate that the high rates of LVI and stage III disease observed alongside TRG 3 in geriatric gastric cancer patients reflect more aggressive tumor biology, and support the role of TRG as an important pathological marker that can be used for prognostic risk stratification in this population. Although perineural invasion (PNI) was numerically more frequent in the poor pathological response group (75.0% versus 58.0%), this difference did not reach statistical significance. This suggests a potential link between PNI and aggressive tumor behavior, but also indicates that the sample size of our cohort and the heterogeneity within the geriatric subpopulation may have been insufficient to demonstrate an independent prognostic effect of PNI. In the literature, studies specifically evaluating the relationship between PNI, TRG, and survival in geriatric patients are limited, and larger cohort studies are needed [17].

Regarding the perioperative chemotherapy regimen, no statistically significant difference in TRG was observed between patients treated with FOLFOX and those treated with FLOT. Current guidelines recommend FLOT as the standard perioperative regimen, and in suitable patients, the addition of immune checkpoint inhibitors to perioperative therapy is being pursued to achieve higher pathological response and survival rates. In elderly patients, however, in addition to regimen efficacy, performance status, comorbidities, and treatment tolerance are critically important factors in treatment selection [13,18]. In our study, although no significant difference in TRG was observed between FLOT and FOLFOX, this finding should not be interpreted as evidence of comparable efficacy between the two regimens. The relatively small number of patients in the FOLFOX group (*n* = 17) limited the statistical power to detect potential differences between the regimens. Moreover, treatment allocation was based on clinical judgment rather than randomization and was influenced by factors such as age, comorbidities, performance status, and anticipated treatment tolerance, introducing potential confounding by indication. Therefore, our study was not designed or adequately powered to compare the efficacy of FLOT and FOLFOX.

In our cohort of older patients with gastric adenocarcinoma, median DFS was 42.4 months in the good pathological response group (TRG 1–2) and 13.0 months in the poor pathological response group (TRG 3). Median OS was 47.7 months in patients with a good pathological response and 15.5 months in those with a poor pathological response. These findings suggest that TRG may be an important parameter for determining long-term prognosis in the geriatric population. In the study by Tomasello et al., OS was significantly longer in patients with a good TRG, and TRG was shown to have prognostic value after neoadjuvant therapy [19]. In a meta-analysis by Hayashi and colleagues assessing the prognostic value of TRG after neoadjuvant chemotherapy in patients with gastric/gastroesophageal adenocarcinoma, the risk of death was significantly lower in patients with good pathological response than in those with poor response (RR 0.53; 95% CI 0.46–0.60; *p* < 0.001). These results, which are mostly derived from younger and more selected patient populations, are consistent with the findings of our study in geriatric patients. In our analysis, the markedly longer median OS in the good pathological response group compared with the poor response group indicates that TRG remains a strong prognostic indicator even in older patients [20].

Similarly, in the study by Lombardi et al., conducted in a more heterogeneous patient population, the median DFS was 52 months in the good response group, whereas it remained at 19 months in the poor response group. In our study focusing on geriatric patients, good pathological response was likewise clearly associated with longer DFS and OS [21]. In our geriatric cohort, Kaplan–Meier analysis showed that DFS and OS were clearly longer in the good pathological response group (TRG 1–2) than in the poor pathological response group (TRG 3). In the multivariable Cox regression analysis, ECOG performance status, lymphovascular invasion, and poor pathological response (TRG 3) remained independent prognostic factors for both DFS and OS. These findings suggest that pathological response provides prognostic information for both DFS and OS in geriatric gastric cancer patients, together with performance status and lymphovascular invasion.

In our study, all analyses were conducted specifically in a geriatric patient population. In the literature, most studies examining the prognostic importance of pathological response after neoadjuvant therapy have been performed in younger and more heterogeneous populations, and data on older patients are limited. In geriatric patients, chronological age alone is insufficient for guiding treatment decisions; physiological reserve, comorbidities, and functional status should be evaluated together. In our real-world cohort, the observation that the strong relationship between pathological response and survival is preserved in appropriately selected geriatric patients receiving neoadjuvant/perioperative therapy suggests that this treatment approach should not be withheld solely on the basis of advanced age. Moreover, these findings support the role of TRG as a valuable prognostic marker in geriatric patients.

All of these results should be interpreted in light of certain limitations. This was a retrospective, single-center study. Because it was conducted in a cohort of geriatric patients with gastric adenocarcinoma receiving neoadjuvant therapy, the sample size was limited, which may have reduced statistical power in subgroup analyses. Furthermore, the study included patients who were able to receive neoadjuvant/perioperative chemotherapy and subsequently undergo curative gastrectomy, and 97.6% of the cohort had an ECOG performance status of 0–1. Therefore, the study population represents a selected group of older patients with relatively good performance status compared with the broader geriatric gastric cancer population. Patients with severe frailty, a high comorbidity burden, or an inability to complete preoperative treatment were underrepresented, which may limit the generalizability of our findings to the broader geriatric population. In addition, standardized frailty scales were not applied to all patients, which may have prevented a full assessment of some parameters related to treatment tolerance and prognosis in older individuals. The use of two different perioperative chemotherapy regimens and the non-randomized treatment allocation may have introduced potential confounding. In addition, the small number of patients treated with FOLFOX limited the ability to make meaningful comparisons between the two regimens. The grouping of TRG 1 and TRG 2 into a single good-response category represents another limitation of our study, as these categories reflect different degrees of residual tumor and may have distinct biological and prognostic implications. Given the small number of patients with TRG 1 (*n* = 5), separate analysis of this subgroup would have had limited statistical reliability. Although TRG 1 and TRG 2 were combined in the primary analyses, the distribution of each TRG category was additionally presented separately in the manuscript. Nevertheless, the fact that the study is based solely on real-world data from geriatric patients with gastric adenocarcinoma and that it evaluates long-term survival outcomes in conjunction with pathological response renders our findings clinically meaningful.

## 5. Conclusions

This study evaluated the prognostic impact of TRG on long-term survival in geriatric patients (≥65 years) with gastric adenocarcinoma treated with neoadjuvant/perioperative therapy. Good pathological response (TRG 1–2) was associated with both DFS and OS, whereas poor pathological response (TRG 3) was identified as an independent prognostic factor for both DFS and OS. In our real-world geriatric cohort, the preservation of a strong relationship between good pathological response and survival after neoadjuvant/perioperative treatment suggests that an appropriately selected treatment approach can be effective and clinically meaningful in older patients. Furthermore, TRG appears to be a valuable prognostic marker in this population, which should be taken into account for post-treatment risk stratification and the planning of follow-up strategies.

## Figures and Tables

**Table 1:** Baseline demographic and clinicopathological characteristics.

Variable	Statistics
Number of patients	82
Age (years) ^#^	69.3 ± 4.8
Sex, *n* (%)	
Male	62 (75.6)
Female	20 (24.4)
Body mass index (kg/m^2^) ^†^	21.5 (17.6–30.4)
ECOG performance status, *n* (%)	
0	36 (43.9)
1	44 (53.7)
2	2 (2.4)
Comorbidities, *n* (%)	
Hypertension	34 (41.5)
Diabetes mellitus	22 (26.8)
Coronary artery disease	15 (18.3)
Chronic kidney disease	3 (3.7)
Other	1 (1.2)
Histopathological diagnosis, *n* (%)	
Well-differentiated adenocarcinoma	11 (13.4)
Moderately differentiated adenocarcinoma	26 (31.7)
Poorly differentiated adenocarcinoma	45 (54.9)
Primary tumor location, *n* (%)	
Antrum	25 (30.5)
Gastroesophageal junction (GEJ)	13 (15.9)
Cardia	32 (39.0)
Corpus	12 (14.6)

^#^ Mean (±standard deviation) ^†^ median (range). Categorical variables are presented as *n* (%). Abbreviations: ECOG, Eastern Cooperative Oncology Group; GEJ, gastroesophageal junction.

**Table 2:** Clinicopathological and treatment characteristics.

Variable	Statistics
Clinical stage, *n* (%)	
II	28 (34.1)
III	54 (65.9)
Type of gastrectomy, *n* (%)	
Total gastrectomy	49 (59.8)
Subtotal gastrectomy	33 (40.2)
Lymphovascular invasion, *n* (%)	57 (69.5)
Perineural invasion, *n* (%)	53 (64.6)
Number of dissected lymph nodes ^†^	22 (10–52)
Tumor regression grade, *n* (%)	
Good pathological response (TRG 1–2)	50 (61.0)
Poor pathological response (TRG 3)	32 (39.0)
Perioperative chemotherapy regimen, *n* (%)	
FLOT	65 (79.3)
FOLFOX	17 (20.7)
Neoadjuvant chemotherapy cycles ^†^	4 (3–6)
Adjuvant chemotherapy cycles ^†^	4 (0–6)
Relapse/progression of disease, *n* (%)	
No	52 (63.4)
Yes	30 (36.6)
Second-line treatment, *n* (%)	17 (56.7)
Death, *n* (%)	44 (53.7)

^†^ Data are presented as median (range). Categorical variables are presented as *n* (%). Abbreviations: TRG, tumor regression grade.

**Table 3:** Comparison of clinicopathological characteristics and clinical outcomes according to pathological response.

Variable	Good Pathological Response (TRG 1–2) (*n* = 50)	Poor Pathological Response (TRG 3) (*n* = 32)	*p*
Age (years) ^#^	68.9 ± 4.3	69.8 ± 5.5	0.653
Sex, *n* (%)			0.341
Male	36 (72.0)	26 (81.3)	
Female	14 (28.0)	6 (18.8)	
Primary tumor location, *n* (%)			0.133
Antrum	19 (38.0)	6 (18.8)	
GEJ	5 (10.0)	8 (25.0)	
Cardia	18 (36.0)	14 (43.8)	
Corpus	8 (16.0)	4 (12.5)	
Type of gastrectomy, *n* (%)			0.386
Total gastrectomy	28 (56.0)	21 (65.6)	
Subtotal gastrectomy	22 (44.0)	11 (34.4)	
Lymphovascular invasion, *n* (%)	28 (56.0)	29 (90.6)	**0.001**
Perineural invasion, *n* (%)	29 (58.0)	24 (75.0)	0.116
Clinical stage, *n* (%)			**<0.001**
II	26 (52.0)	2 (6.3)	
III	24 (48.0)	30 (93.8)	
Perioperative chemotherapy regimen, *n* (%)			0.361
FLOT	38 (76.0)	27 (84.4)	
FOLFOX	12 (24.0)	5 (15.6)	
Disease progression, *n* (%)			**0.044**
No	36 (72.0)	16 (50.0)	
Yes	14 (28.0)	16 (50.0)	
Death, *n* (%)			**0.002**
No	30 (60.0)	8 (25.0)	
Yes	20 (40.0)	24 (75.0)	

^#^ Mean (±standard deviation). Categorical variables are presented as *n* (%). Bold values indicate statistical significance (*p* < 0.05).

**Table 4:** Survival outcomes according to pathological response.

Variable	Good Pathological Response (TRG 1–2)	Poor Pathological Response (TRG 3)	Overall	*p* Value
Median DFS (95% CI), months	42.4 (29.3–56.1)	13.0 (9.9–16.0)	27.1 (21.1–33.1)	**<0.001**
Median OS (95% CI), months	47.7 (26.2–69.2)	15.5 (9.6–21.4)	27.0 (21.3–32.7)	**<0.001**

Abbreviations: DFS, disease-free survival; OS, overall survival; CI, confidence interval; TRG, tumor regression grade. Bold values indicate statistical significance (*p* < 0.05).

**Table 5:** Univariable and multivariable Cox regression analysis for DFS.

Variable	Univariable HR (95% CI)	*p*	Multivariable HR (95% CI)	*p*
ECOG performance status	3.102 (1.601–6.012)	**<0.001**	2.697 (1.381–5.269)	**0.004**
Clinical stage (III vs. II)	2.188 (1.078–4.440)	**0.030**	0.836 (0.364–1.921)	0.673
Lymphovascular invasion	6.433 (2.271–18.220)	**<0.001**	4.960 (1.545–15.919)	**0.007**
Pathological response (TRG 3 vs. TRG 1–2)	2.893 (1.581–5.296)	**<0.001**	2.066 (1.055–4.045)	**0.034**

Abbreviations: HR, hazard ratio; CI, confidence interval; ECOG, Eastern Cooperative Oncology Group; TRG, tumor regression grade. Bold values indicate statistical significance (*p* < 0.05).

**Table 6:** Univariable and multivariable Cox regression analysis for OS.

Variable	Univariable HR (95% CI)	*p*	Multivariable HR (95% CI)	*p*
ECOG performance status	3.106 (1.613–5.980)	**<0.001**	2.719 (1.398–5.290)	**0.003**
Clinical stage (III vs. II)	2.346 (1.144–4.812)	**0.020**	0.954 (0.412–2.213)	0.914
Lymphovascular invasion	6.341 (2.248–17.885)	**<0.001**	4.448 (1.400–14.132)	**0.011**
Pathological response (TRG 3 vs. TRG 1–2)	3.239 (1.737–6.040)	**<0.001**	2.311 (1.161–4.601)	**0.017**

Abbreviations: HR, hazard ratio; CI, confidence interval; ECOG, Eastern Cooperative Oncology Group; TRG, tumor. Bold values indicate statistical significance (*p* < 0.05).

## Data Availability

The datasets generated and/or analyzed during the current study are available from the corresponding author on reasonable request.

## Abbreviations

BMI	Body mass index
CI	Confidence interval
DFS	Disease-free survival
ECF	Epirubicin, cisplatin, and fluorouracil
ECX	Epirubicin, cisplatin, and capecitabine
ECOG	Eastern Cooperative Oncology Group
FLOT	Fluorouracil, leucovorin, oxaliplatin, and docetaxel
FOLFOX	Fluorouracil, leucovorin, and oxaliplatin
GEJ	Gastroesophageal junction
HR	Hazard ratio
LVI	Lymphovascular invasion
OS	Overall survival
pCR	Pathological complete response
PNI	Perineural invasion
TRG	Tumor regression grade
